# Pilloin, A Flavonoid Isolated from *Aquilaria sinensis*, Exhibits Anti-Inflammatory Activity In Vitro and In Vivo

**DOI:** 10.3390/molecules23123177

**Published:** 2018-12-02

**Authors:** Yun-Chen Tsai, Sin-Ling Wang, Mei-Yao Wu, Chia-Huei Liao, Chao-Hsiung Lin, Jih-Jung Chen, Shu-Ling Fu

**Affiliations:** 1 Institute of Traditional Medicine, National Yang-Ming University, Taipei 11221, Taiwan; tyc202006@gmail.com (Y.-C.T.); sweet791020@gmail.com (C.-H.L.); 2 School of Pharmacy, College of Pharmacy, Kaohsiung Medical University, Kaohsiung 80708, Taiwan; s8332805@yahoo.com.tw; 3 Research Center for Traditional Chinese Medicine, Department of Medical Research, China Medical University Hospital, Taichung 40402, Taiwan; meiyao19790919@yahoo.com.tw; 4 Department of Life Sciences and Institute of Genome Sciences, National Yang-Ming University, Taipei 11221, Taiwan; chlin2@ym.edu.tw; 5 Faculty of Pharmacy, National Yang-Ming University, Taipei 11221, Taiwan; 6 Department of Medical Research, China Medical University Hospital, China Medical University, Taichung 40447, Taiwan

**Keywords:** pilloin, flavonoid, *Aquilaria sinensis*, anti-inflammation, NF-κB

## Abstract

Flavonoids, widely present in medicinal plants and fruits, are known to exhibit multiple pharmacological activities. In this study, we isolated a flavonoid compound, pilloin, from *Aquilaria sinensis* and investigated its anti-inflammatory activity in bacterial lipopolysaccharide-induced RAW 264.7 macrophages and septic mice. Pilloin inhibited NF-κB activation and reduced the phosphorylation of IκB in LPS-stimulated macrophages. Moreover, pilloin significantly suppressed the production of pro-inflammatory molecules, such as TNF-α, IL-6, COX-2 and iNOS, in LPS-treated RAW 264.7 macrophages. Additionally, pilloin suppressed LPS-induced morphological alterations, phagocytic activity and ROS elevation in RAW 264.7 macrophages. The mitogen-activated protein kinase-mediated signalling pathways (including JNK, ERK, p38) were also inhibited by pilloin. Furthermore, pilloin reduced serum levels of TNF-α (from 123.3 ± 7 to 46.6 ± 5.4 ng/mL) and IL-6 levels (from 1.4 ± 0.1 to 0.7 ± 0.1 ng/mL) in multiple organs of LPS-induced septic mice (liver: from 71.8 ± 3.2 to 36.7 ± 4.3; lung: from 118.6 ± 10.6 to 75.8 ± 11.9; spleen: from 185.9 ± 23.4 to 109.6 ± 18.4; kidney: from 160.3 ± 11.8 to 75 ± 10.8 pg/mL). In summary, our results demonstrate the anti-inflammatory potential of pilloin and reveal its underlying molecular mechanism of action.

## 1. Introduction

Inflammation, which is triggered by infection and injury, is an important process in the host defence system and homeostasis [1]. Upon pathogen recognition, macrophages are stimulated to produce pro-inflammatory molecules, such as tumor necrosis factor-alpha (TNF-α), nitric oxide (NO), inducible nitric oxide synthase (iNOS), cyclooxygenase (COX)-2, and reactive oxygen species (ROS) to fight pathogens [2,3,4]. However, persistent inflammation is highly associated with a variety of diseases, such as rheumatism, sepsis, diabetes and cancer [3,5]. For example, sepsis caused by severe bacterial infection is a prevalent life-threatening disease that can lead to tissue damage, organ failure and/or death due to dysregulated inflammatory responses [6].

Nuclear factor κB (NF-κB) is an essential transcription factor that regulates genes involved in multiple physiological processes, such as immunity, inflammation and apoptosis [7,8,9]. The IκB kinase (IKK) complex regulates NF-κB activity through regulation of the IκB protein. The phosphorylation of IκB by IKK leads to its degradation via the proteosome, which results in the translocation of NF-κB from the cytoplasm to the nucleus, the binding of NF-κB to its responsive sequences, and the transactivation of NF-κB downstream target genes [7]. The activation of NF-κB subsequently leads to the production of inflammatory molecules (e.g., TNF-α, interleukin-6 (IL-6), NO, iNOS and COX-2), as well as the activation and recruitment of innate immune cells [10,11]. Therefore, NF-κB is an ideal target for the development of anti- inflammatory drugs.

Plant-derived natural products play a significant role in drug development. Many anti-inflammatory natural compounds, including fisetin, curcumin, puerarin and andrographolide, suppress NF-κB activity under inflammatory conditions [12,13,14,15]. Flavonoids are widely present in plants and are common ingredients in fruits, herbal medicines and teas. Accumulating studies have demonstrated that flavonoids exhibit multiple pharmacological activities, such as neuroprotection, cardiovascular protection and prevention of metabolic diseases [16]. Pilloin (3′,5-dihydroxy-4′,7-dimethoxyflavone) is a flavonoid present in foods and medicinal plants, such as *Aquilaria sinensis*, *Piper auritum*, *Murraya panaculata* and *Daphne aurantiaca* [17,18,19,20]. In this study, we isolated pilloin from *A. sinensis* by activity-guided fractionation. Because the anti-inflammatory effect of pilloin has rarely been described, we investigated its anti-inflammatory activity in bacterial lipopolysaccharide (LPS)-induced RAW 264.7 macrophages and septic mice.

## 2. Results

### 2.1. Isolation and Identification of Pilloin from A. sinensis

**Figure 1:**
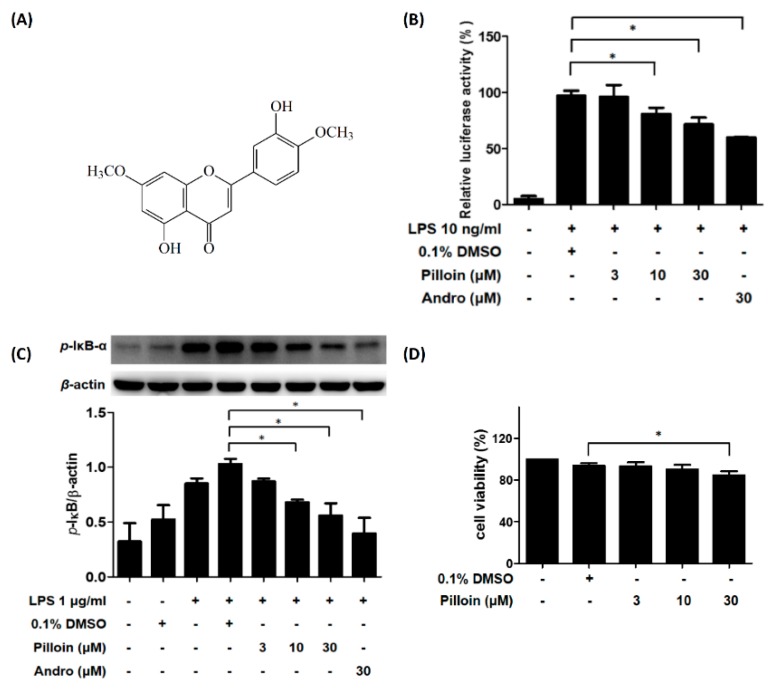
Pilloin inhibits NF-κB activity in LPS-stimulated RAW 264.7 macrophages. (**A**) Structure of pilloin (3′,5-dihydroxy-4′,7-dimethoxyflavone). (**B**) Pilloin inhibits NF-κB activity in a concentration-dependent manner. RAW 264.7/Luc-P1 macrophages (4 × 10^5^ cells in MP-24 plates) were treated with pilloin or 0.1% DMSO for 1 h, followed by LPS (10 ng/mL) for 6 h. (**C**) Pilloin reduces LPS-induced IκB phosphorylation. RAW 264.7 macrophages (10^6^ cells in MP-6 plates) were treated with pilloin or 0.1% DMSO for 6 h, followed by LPS (1 μg/mL) for 30 min. IκB phosphorylation and β-actin expression were examined by Western blot analysis. β-actin served as the loading control. (**D**) Pilloin shows low cytotoxicity on RAW 264.7 macrophages. RAW 264.7 cells (2 × 10^4^ cells in MP-96 plates) were treated with pilloin or 0.1% DMSO for 24 h and subjected to the MTT assay. 0.1% DMSO served as the vehicle control. Andro (andrographolide) served as the positive control. * indicates a significant difference versus control groups (*p* < 0.05).

The EtOAc-soluble fractions (MeOH extract) from stem barks of *A. sinensis* were purified using a silica gel column and preparative thin layer chromatography (TLC), which yielded approximately 28.5 mg of pilloin. Its molecular weight was determined on the basis of the positive ESI-MS at *m*/*z* 315 [M + H] (Supplementary Figure S1) and the structure was identified by the ^1^H-NMR data (Supplementary Figure S2). The structure was also confirmed by comparison of the UV and IR data of the isolated pilloin with those from the literature (Figure 1A) [21,22].

### 2.2. Pilloin Suppresses NF-κB Activity in LPS-Induced RAW 264.7 Macrophages

LPS-activated macrophages serve as an in vitro system to study inflammation [12]. Our laboratory previously established an LPS-responsive macrophage cell clone, RAW 264.7/Luc-P1, in which the activity of NF-κB correlates with the expression of the reporter gene (*luciferase*) upon LPS treatment [23]. We applied this cell line to evaluate the effects of pilloin on LPS-stimulated NF-κB activity in RAW 264.7 macrophages. As shown in Figure 1B, pilloin inhibited NF-κB activation in a concentration-dependent manner. Consistent with this observation, the phosphorylation of IκB (a negative regulator of NF-κB) was also reduced by pilloin in LPS-stimulated macrophages in a concentration-dependent manner (Figure 1C). Pilloin did not cause cytotoxicity at 3 or 10 μM, and only caused slight toxicity at 30 μM (85% viability), indicating pilloin has considerably low cytotoxicity (Figure 1D).

### 2.3. Pilloin Suppresses the Production of Pro-Inflammatory Molecules Induced by LPS

**Figure 2:**
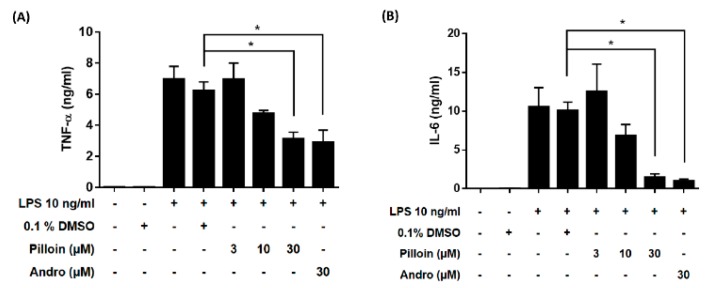
Pilloin suppresses the production of pro-inflammatory cytokines in LPS-stimulated RAW 264.7 macrophages. RAW 264.7 macrophages (2 × 10^5^ in MP-96 plates) were treated with pilloin or 0.1% DMSO for 1 h, followed by LPS for 24 h. Culture medium was then assayed for (**A**) TNF-α and (**B**) IL-6 production using ELISA. * indicates a significant difference versus the LPS-treated vehicle control (*p* < 0.05).

**Figure 3:**
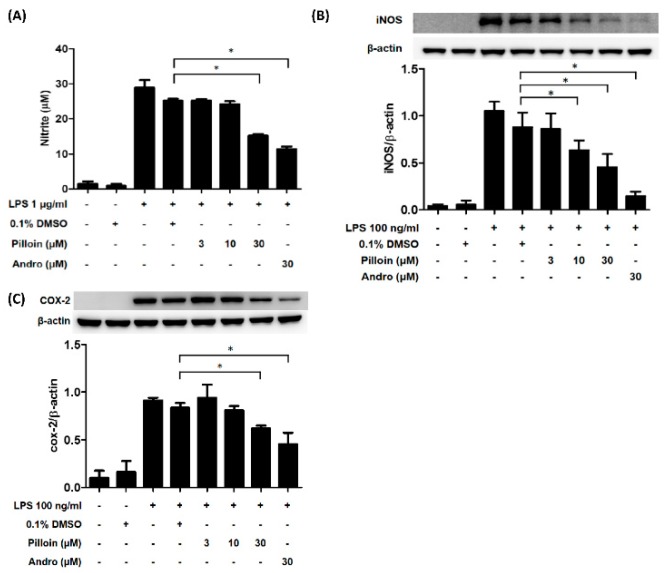
Pilloin reduces the production of NO, iNOS and COX-2 in LPS-stimulated RAW 264.7 macrophages. (**A**) Pilloin inhibits LPS-stimulated NO production in RAW 264.7 macrophages. RAW 264.7 macrophages (4 × 10^4^ cells in MP-96 plates) were treated with pilloin or 0.1% DMSO for 1 h, followed by LPS (1 μg/mL) for 24 h. Culture medium was assayed for NO production using the Griess assay. Pilloin inhibits iNOS (**B**) and COX-2 (**C**) expression induced by LPS. In (**B**,**C**), the RAW 264.7 macrophages (5 × 10^5^ cells in MP-6 plates) were treated with pilloin or 0.1% DMSO for 1 h, followed by LPS (100 ng/mL) for 24 h. The expression of iNOS, COX-2 and β-actin in treated cells was examined by Western blot analysis. β-actin served as the internal control. Quantification data from three independent experiments are shown. 0.1% DMSO served as the vehicle control. Andro (andrographolide) served as the positive control. * indicates a significant difference versus the LPS-treated group (*p* < 0.05).

Under LPS stimulation, NF-κB activation in macrophages results in the production of pro-inflammatory molecules, such as TNF-α, IL-6 and NO [7,10]. We next determined whether pilloin affects the production of pro-inflammatory molecules in LPS-induced macrophages. As shown in Figure 2, pilloin reduced TNF-α and IL-6 production at 30 μM, as measured by ELISA. Pilloin also decreased NO production and iNOS expression relative to the vehicle group (Figure 3A,B). The expression of COX-2, a downstream pro-inflammatory enzyme of NF-κB, was also suppressed by pilloin at 30 μM (Figure 3C).

### 2.4. Pilloin Suppresses LPS-Induced Morphological Alterations, Phagocytic Activity and ROS Elevation in RAW 264.7 Macrophages

**Figure 4:**
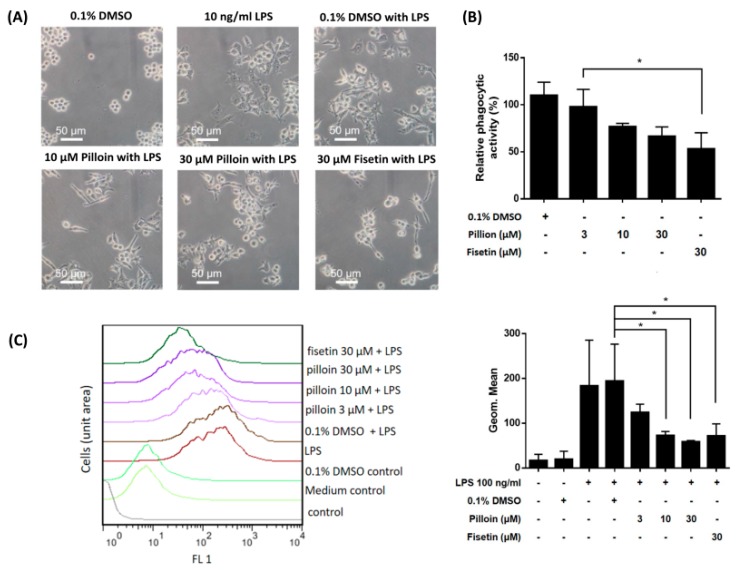
Pilloin suppresses morphological alterations and decreases phagocytic activity in LPS-stimulated RAW 264.7 macrophages. (**A**) Pilloin attenuates morphological alterations induced by LPS. RAW 264.7 macrophages (10^5^ cells in MP-6 plates) were treated with pilloin or 0.1% DMSO for 1 h, followed by LPS (10 ng/mL) for 24 h. Images were taken under a phase-contrast microscope. (**B**) Pilloin reduces the phagocytic activity of macrophages. RAW 264.7 macrophages (2 × 10^5^ cells in MP-24 plates) were treated with pilloin or 0.1% DMSO for 24 h, and the fluorescence intensity (480 nm/520 nm) was detected using Infinite^®^ 200 PRO. The relative phagocytic activity of vehicle and pilloin-treated RAW 264.7 cells was determined based on their fluorescence intensity versus that of untreated cells. (**C**) Pilloin inhibits ROS generation induced by LPS. RAW 264.7 macrophages (5 × 10^5^ cells in MP-6 plates) were treated with pilloin or 0.1% DMSO for 1 h, followed by LPS (100 ng/mL) for 24 h. Fisetin served as the positive control. * indicates a significant difference versus the control group (*p* < 0.05).

Activated macrophages typically display a distinct morphology and enhanced phagocytic activity [23,24]. As shown in Figure 4A, vehicle-treated RAW 264.7 cells were round and refractive, while LPS-treated cells were polygonal and more adherent. Notably, pilloin treatment attenuated LPS-induced morphological changes. Furthermore, the phagocytic activity of RAW 264.7 was reduced by pilloin (Figure 4B). LPS-activated macrophages exhibit elevated ROS. As shown in Figure 4C, ROS levels in LPS-treated macrophages were diminished by pilloin in a concentration-dependent manner. Together, the above results (Figure 1, Figure 2, Figure 3 and Figure 4) suggest that pilloin exhibits anti-inflammatory activity via attenuating LPS-induced macrophage activation.

### 2.5. Pilloin Inhibits Mitogen-Activated Protein Kinase (MAPK)-Mediated Signalling Pathways

**Figure 5:**
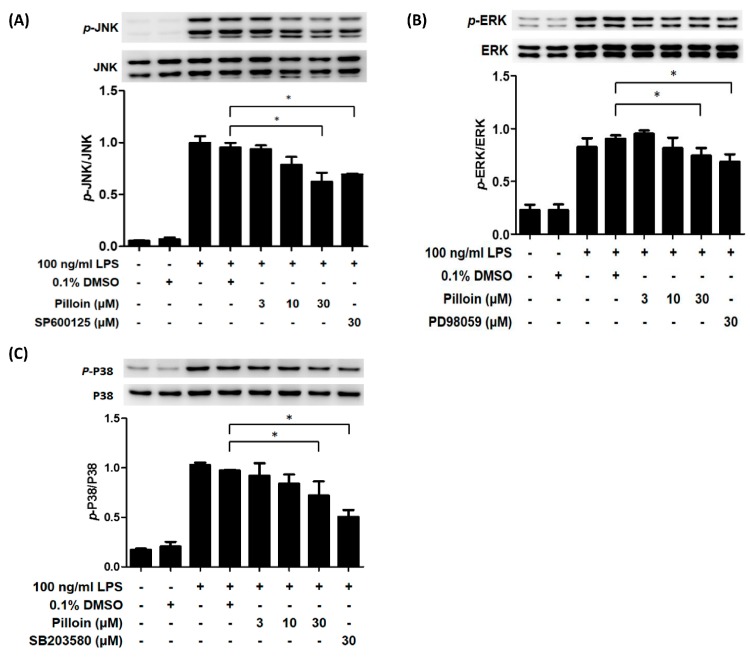
Effects of pilloin on MAPK signalling pathways in LPS-stimulated RAW 264.7 macrophages. RAW 264.7 cells (10^6^ cells in MP-6 plates) were treated with vehicle, pilloin or the indicated inhibitor for 6 h before incubation with LPS (100 ng/mL) for 20 min. Cell lysates were analysed by Western blot to detect the activation of (**A**) JNK, (**B**) ERK and (**C**) p38. PD98059 (ERK inhibitor), SB203580 (p38 inhibitor) and SP600125 (JNK inhibitor) served as positive controls. * indicates a significant difference versus the LPS-treated group (*p* < 0.05).

MAPKs are also involved in LPS-induced pro-inflammatory responses [25]. Therefore, we measured the effect of pilloin on the activation status of MAPKs. Expression of both the total and active forms of MAPK family proteins, JNK, ERK and p38, in pilloin-pretreated cells were detected by Western blot analysis. As shown in Figure 5, pilloin at 30 μM suppressed the activation of all MAPK signalling pathways in LPS-activated macrophages.

### 2.6. Pilloin Attenuates LPS-Induced Cytokine Expression In Vivo

**Figure 6:**
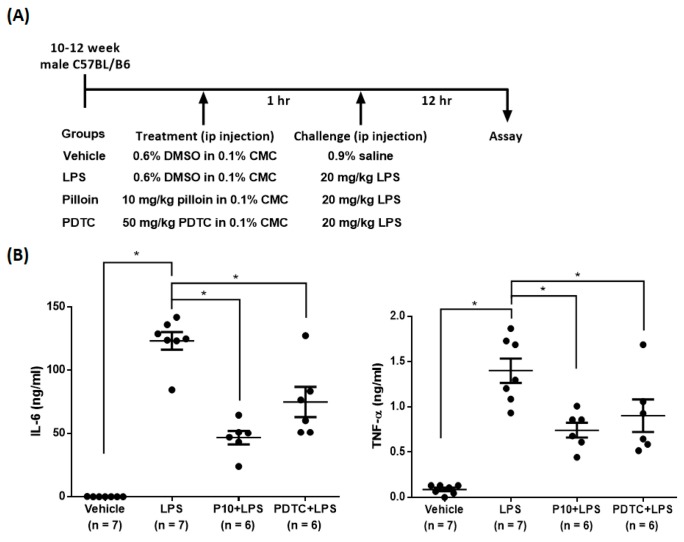
Pilloin reduces the serum level of pro-inflammatory cytokines in LPS-induced septic mice. (**A**) Experimental scheme for LPS-induced sepsis in mice and drug treatment. (**B**) Pilloin reduces the levels of pro-inflammatory cytokines in the serum of LPS-treated mice. Data were collected from mice exposed to various treatments for 12 h after LPS challenge. The production of TNF-α and IL-6 was determined by ELISA respectively. The values are shown as mean ± SEM. * indicates statistically significant (*p* < 0.05). CMC: carboxymethyl cellulose.

**Figure 7:**
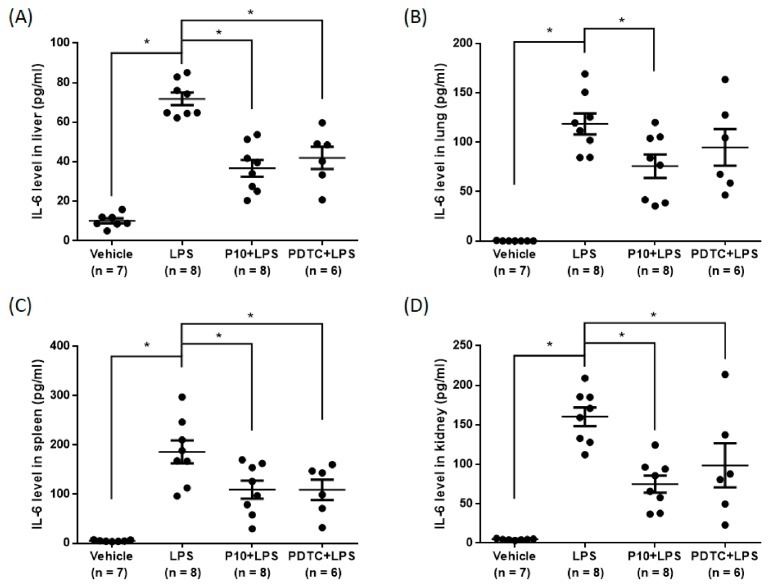
Pilloin decreases tissue IL-6 levels in LPS-induced septic mice. Pilloin decreases the levels of IL-6 in the liver (**A**), lung (**B**), spleen (**C**) and kidney (**D**) of LPS-treated mice. Experimental scheme is described in Figure 6A, and data were collected from mice exposed to various treatments for 12 h after LPS challenge. The production of IL-6 was determined by ELISA. The values were shown as mean ± SEM. * indicates statistically significant (*p* < 0.05).

Sepsis caused by the complicated interactions between pathogens and the host immune system results in a cytokine storm [26]. Previous studies have demonstrated that LPS stimulates the production of pro-inflammatory cytokines, such as IL-6 and TNF-α [26,27]. Therefore, we evaluated the effects of pilloin on TNF-α and IL-6 levels in an LPS-induced sepsis model. As shown in Figure 6, mice treated with pilloin (10 mg/kg) exhibited decreased serum TNF-α and IL-6 levels compared with vehicle-treated LPS mice. Moreover, pilloin reduced IL-6 production in liver, lung, spleen and kidney (Figure 7). The serum levels of glutamate oxaloacetate transaminase (GOT), glutamate pyruvate transaminase (GPT) and creatinine (CRE) are close to those of control groups, indicating pilloin does not affect renal and liver function (Supplementary Figure S3).

## 3. Discussion

In this study, the anti-inflammatory potential of pilloin was demonstrated in LPS-activated macrophages and in LPS-induced septic mouse model. Pilloin inhibited NF-κB and MAPK signalling pathways in LPS-activated macrophages (Figure 1 and Figure 5). Pro-inflammatory cytokines (e.g., TNF-α and IL-6), as well as enzymes (e.g., iNOS and COX-2) were also downregulated by pillion (Figure 2 and Figure 3). In addition, the phenotypes and functions of activated macrophages (i.e., ROS production and phagocytic activity) were also suppressed by pillion (Figure 4). Furthermore, pilloin attenuated the LPS-stimulated production of cytokines (i.e., TNF-α and IL-6) in serum and in tissues in vivo (Figure 6 and Figure 7). Although previous studies have described that pilloin reduced NO production in mouse peritoneal macrophages and decreased ROS generation in rabbit neutrophils [28,29], our study fully explored the anti-inflammatory activity of pilloin both in vitro and in vivo. Our study is also the first to describe the in vivo efficacy and the molecular mechanism of pilloin-mediated anti-inflammatory activity.

According to previous studies, stimulation of TLR4 by LPS in macrophages triggers myeloid differentiation factor 88-dependent (MyD88) and the MyD88-independent signalling pathways [30,31,32,33]. In MyD88-dependent signalling pathway, activation of tumor necrosis factor-receptor- associated factor 6 (TRAF6) results in the activation of nuclear factor-κB (NF-κB) through phosphorylation of IκB via IκB kinase (IKK), as well as the activation of MAPK (ERK, p38 and JNK) which subsequently leads to activation of AP-1 transcription factors. As a consequence, activation of NF-κB and AP-1 induces the expression of downstream pro-inflammatory molecules such as iNOS, COX-2, TNF-α, IL-6 [30,31,32,33]. Our data showed that pilloin inhibited IκB phosphorylation and suppressed the transcriptional activity of NF-κB in LPS-treated macrophages (Figure 1). In addition, the known downstream targets of NF-κB pathways, such as TNF-α, IL-6, iNOS and COX-2 were all suppressed upon pilloin treatment (Figure 2 and Figure 3), suggesting the NF-κB pathway as an essential pathway underlying the anti-inflammatory effects of pilloin. On the other hand, the activities of MAPKs as well as its downstream molecules (iNOS, TNF-α and ROS) were correspondingly suppressed by pilloin (Figure 2, Figure 3, Figure 4C and Figure 5), indicating the possibility that MAPK pathway is another target of pilloin. Since both NF-κB and MAPKs are downstream of LPS-mediated TLR4 signalling pathway, it is possible that pilloin acts on an upstream regulator of LPS-mediated signalling pathway, which leads to simultaneous inhibition of both NF-κB and MAPK pathways. Alternatively, there might be a sequential relationship between the inhibition of MAPK and NF-κB based on current publications [34,35,36]. Certainly, the direct molecular targets of pilloin and the detailed molecular mechanism of pillion-mediated anti-inflammatory effects merit further investigation.

According to previous studies, stimulation of TLR4 by LPS in macrophages triggers myeloid differentiation factor 88-dependent (MyD88) and the MyD88-independent signalling pathways [30,31,32,33]. In MyD88-dependent signalling pathway, activation of tumor necrosis factor-receptor- associated factor 6 (TRAF6) results in the activation of nuclear factor-κB (NF-κB) through phosphorylation of IκB via IκB kinase (IKK), as well as the activation of MAPK (ERK, p38 and JNK) which subsequently leads to activation of AP-1 transcription factors. As a consequence, activation of NF-κB and AP-1 induces the expression of downstream pro-inflammatory molecules such as iNOS, COX-2, TNF-α, IL-6 [30,31,32,33]. Our data showed that pilloin inhibited IκB phosphorylation and suppressed the transcriptional activity of NF-κB in LPS-treated macrophages (Figure 1). In addition, the known downstream targets of NF-κB pathways, such as TNF-α, IL-6, iNOS and COX-2 were all suppressed upon pilloin treatment (Figure 2 and Figure 3), suggesting the NF-κB pathway as an essential pathway underlying the anti-inflammatory effects of pilloin. On the other hand, the activities of MAPKs as well as its downstream molecules (iNOS, TNF-α and ROS) were correspondingly suppressed by pilloin (Figure 2, Figure 3, Figure 4C and Figure 5), indicating the possibility that MAPK pathway is another target of pilloin. Since both NF-κB and MAPKs are downstream of LPS-mediated TLR4 signalling pathway, it is possible that pilloin acts on an upstream regulator of LPS-mediated signalling pathway, which leads to simultaneous inhibition of both NF-κB and MAPK pathways. Alternatively, there might be a sequential relationship between the inhibition of MAPK and NF-κB based on current publications [34,35,36]. Certainly, the direct molecular targets of pilloin and the detailed molecular mechanism of pillion-mediated anti-inflammatory effects merit further investigation.

Sepsis is characterized by a cytokine storm [37]. It has been reported that the plasma levels of TNF-α are increased in sepsis patients and in animal models [26]. Furthermore, previous studies have demonstrated that IL-6 is a crucial cytokine in the pathophysiology of severe sepsis and that increased levels of IL-6 are related with the highest risk of death in sepsis patients [17]. Our data, for the first time, demonstrated that pilloin reduces IL-6 and TNF-α levels in an LPS-induced sepsis model (Figure 6 and Figure 7). Furthermore, the values of serum hepatic and renal biomarkers in pilloin-treated mice are within normal ranges, indicating pilloin does not cause toxicity (Supplementary Figure S3). Together, our data support the anti-sepsis potential of pilloin.

Flavonoids exert diverse biological activities such as anti-inflammation and anti-oxidation. The crucial structural features underlying the anti-inflammatory activities of flavonoids are the unsaturation in the C ring, the carbonyl group at C-4 and the number and position of the hydroxyl groups. Flavonoids containing hydroxyl groups at C-5 and C-7 in the aromatic A ring or at C-3′ and C-4′ in the B aromatic ring positions exhibit higher inflammatory activities [38]. Pilloin contains two hydroxyl groups at C-3′ and C-5, consistent with its anti-inflammatory potential observed herein. Currently, there is no pharmacokinetic study on pilloin. It has been reported that methylation of the free hydroxyl groups of flavonoids increases metabolic stability and augments intestinal absorption and oral bioavailability [39]. Since pilloin contains two methoxy groups at C4′ and C7, we speculate that this compound may display fair bioavailability, but this aspect should be further investigated.

Many anti-inflammatory drugs, such as corticosteroids and nonsteroidal anti-inflammatory drugs, have been clinically used to attenuate inflammatory and autoimmune diseases. However, they all cause serious side effects [13]. Therefore, finding other anti-inflammatory compounds is important. Our study demonstrated that pilloin is a potential anti-inflammatory compound both in vitro and in vivo. Previous studies have shown that pilloin exhibited anti-diabetic potency by inhibiting the formation of advanced glycation end products (AGEs) and exerted cytotoxicity on transformed lymphoblasts [28,40]. Therefore, pilloin displays multiple biological functions, and can be further developed as a nutraceutical or pharmaceutical agent.

## 4. Materials and Methods

### 4.1. Chemicals and Antibodies

Lipopolysaccharide (LPS) was purchased from InvivoGen (San Diego, CA, USA). Andrographolide and fisetin were obtained from Sigma-Aldrich (St. Louis, MO, USA). MAPK inhibitors, including PD98059, SB203580, and SP600125, were from Calbiochem (La Jolla, CA, USA). Antibodies against phospho-IkB-α (Ser32), IkB-α, COX-2, Erk, phospho-Erk1/2 (Thr202/Tyr204), JNK, phospho-JNK (Thr183/Tyr185), p38, and phospho-p38 (Thr180/Tyr182) were from Cell Signaling Technology (Danvers, MA, USA). The anti-iNOS and anti-β-actin antibody were obtained from Abcam (Cambridge, UK) and Sigma-Aldrich respectively.

### 4.2. General Experimental Instruments

Melting point was determined using a Yanaco micro-melting point apparatus (Yanaco Co., Ltd., Kyoto, Japan). The infrared (IR) spectrum (KBr) was obtained using a 2000 FT-IR spectrometer (Perkin Elmer, Norwalk, CT, USA). The ultraviolet (UV) spectrum was measured with a UV-240 spectrophotometer (Jasco, Tokyo, Japan). The proton nuclear magnetic resonance (^1^H-NMR) spectra were acquired using a Varian Inova 500 (Varian Inc., Palo Alto, CA, USA) operating at a 500-MHz frequency. Electrospray ionization (ESI)-mass spectra were performed in a positive ion mode using an APEX II mass spectrometer (Bruker, Billerica, MA, USA). Silica gel 60 F-254 (Merck, Darmstadt, Germany) was used for preparative thin-layer chromatography (Prep TLC) and TLC. Column chromatography was followed out using Silica gel 60 (70–230, 230–400 mesh; Merck).

### 4.3. Extraction and Isolation of Pilloin from A. sinensis

Pilloin was isolated and provided by Dr. Jih-Jung Chen (Faculty of Pharmacy at National Yang-Ming University, Taipei, Taiwan). The stem barks of *A. sinensis* were collected from Pingtung City, Taiwan. The extraction and isolation procedures were carried out as described formerly [41]. In brief, the dried stem barks (4.1 kg) of *A. sinensis* were crumbled and extracted 3 times with MeOH (20 L each) for 3 days. The MeOH extracts were concentrated under vacuum at 35 °C, and the residue (390 g) was partitioned between H_2_O and *n*-hexane (1:1). The *n*-hexane layer was concentrated to afford a residue (fraction A, 93 g). The H_2_O layer was extracted with EtOAc, and the EtOAc-soluble part (fraction B, 75 g) and the H_2_O-soluble part (fraction C, 212 g) were segregated. Fraction B (75 g) was chromatographed on silica gel (70–230 mesh, 3.2 kg) and eluted with *n*-hexane, gradually increasing the polarity with acetone or MeOH to obtain 11 fractions: B1 (*n*-hexane/acetone, 20:1, 2 L), B2 (*n*-hexane/acetone, 15:1, 2 L), B3 (*n*-hexane/acetone, 10:1, 6.5 L), B4 (*n*-hexane/acetone, 8:1, 5 L), B5 (*n*-hexane/acetone, 5:1, 10.5 L), B6 (*n*-hexane/acetone, 4:1, 2 L), B7 (*n*-hexane/acetone, 3:1, 12 L), B8 (*n*-hexane/acetone, 2:1, 5 L), B9 (*n*-hexane/acetone, 1:1, 4 L), B10 (acetone, 3 L), and B11 (MeOH, 1 L). Fraction B7 (8.3 g) was chromatographed on silica gel (230–400 mesh, 380 g) and eluted with CH_2_Cl_2_/MeOH (20:1–0:1) to afford 11 fractions (each 1.2 L, B7-1–B7-11). Fraction B7–7 (220 mg) was purified further by preparative TLC (silica gel, CH_2_Cl_2_/EtOAc, 10:1) to afford pilloin (28.5 mg) (Rf = 0.76). The structure of pilloin (3′,5-dihydroxy-4′,7-dimethoxyflavone) is shown in Figure 1A.

### 4.4. Physical and Spectroscopic Data of Pilloin

Yellowish needles (CH_2_Cl_2_-MeOH); m.p. 235–237 °C; UV (MeOH) λ_max_ (logε) 269 (4.15), 335 (4.25) nm; IR (KBr) ν_max_ 3361 (OH), 1652 (C=O) cm^−1^; ^1^H-NMR (CDCl_3_, 500 MHz) δ 3.89 (3H, s, OMe-7), 3.99 (3H, s, OMe-4′), 5.74 (1H, br s, D_2_O exchangeable, OH-3′), 6.37 (1H, d, *J* = 1.5 Hz, H-6), 6.49 (1H, d, *J* = 1.5 Hz, H-8), 6.57 (1H, s, H-3), 6.96 (1H, d, *J* = 8.5 Hz, H-5′), 7.44 (1H, dd, *J* = 8.5, 2.0 Hz, H-6′), 7.47 (1H, d, *J* = 2.0 Hz, H-2′). ESI-MS *m/z* 315 [M + H]^+^. The purity of pillion was greater than 98% based on the ESI-MS and ^1^H-NMR analyses (Supplementary Figures S1 and S2).

### 4.5. Cell Culture

RAW 264.7 murine macrophages were purchased from the Food Industry Research and Development Institute (Hsinchu, Taiwan). The RAW 264.7/Luc-P1, expressing a reporter gene (pELAM1-Luc), is an LPS-responsive cell line generated previously [23]. Both cell lines were cultured in DMEM containing 10% heat-inactivated BCS (Sigma-Aldrich), 100 μg/mL streptomycin, 100 units/mL penicillin, 2 mM l-glutamine and 1 mM sodium pyruvate (Gibco Life Technologies, Grand Island, NY, USA) at 37 °C in a 5% CO_2_ incubator.

### 4.6. Luciferase Reporter Assay

RAW 264.7/Luc-P1 cells (4 × 10^5^ cells in 24-well plates) were treated with pilloin, andrographolide (positive control [14,15]) or vehicle (0.1% DMSO) for 1 h followed by LPS for 6 h, collected, and analysed using luciferase assays. Cell lysates (20 μL) were mixed with 100 μL luciferin (Promega, Madison, WI, USA) immediately prior to luminescence detection [12]. Luminescence was measured on an Infinite^®^ 200 PRO (Tecan Group Ltd., Männedorf, Switzerland).

### 4.7. Enzyme-Linked Immunosorbent Assay (ELISA)

RAW 264.7 cells (2 × 10^5^ cells in 24-well plates) were treated with pilloin, vehicle (0.1% DMSO) or andrographolide (positive control) for 1 h, followed by LPS (10 ng/mL) for 24 h. For measurements of serum and tissue cytokines, blood samples and supernatant of the homogenized tissues were collected from treated mice as described previously [13]. The levels of TNF-α and IL-6 in the medium of cultured RAW 264.7 macrophages, in the serum and in tissue extracts were measured by ELISA (eBioscience, San Diego, CA, USA). A450 nm and A550 nm (reference absorbance) were determined on a Model 680 Microplate Reader (Bio-Rad Laboratories, Hercules, CA, USA).

### 4.8. Determination of NO Production

NO secretion was determined indirectly by measuring nitrite (Griess assay) [42]. RAW 264.7 cells (4 × 10^4^ cells in 96-well plates) were treated with pilloin, vehicle (0.1% DMSO) or andrographolide (positive control) for 1 h, followed by LPS (1 μg/mL) for 24 h. The culture medium was incubated with Griess reagent (1% sulphanilamide, 0.1% *N*-(1-naphthyl) ethylenediamine dihydrochloride in 2.5% H_3_PO_4_) at room temperature for 15 min. A550 nm was recorded using a Model 680 Microplate Reader (Bio-Rad Laboratories). Linear regression was used to determine NO concentration from the standard curve.

### 4.9. MTT Assay

RAW 264.7 cells (2 × 10^4^ cells in 96-well plates) were treated with vehicle (0.1% DMSO) or pilloin for 24 h. Cells were then cultured in medium with 3-(4,5)-dimethylthiahiazo-(z-y1)-3,5-di-phenytetrazoliumromide (MTT) reagent (0.5 mg/mL) for 2 h and incubated with solubilisation buffer (12.5% sodium dodecyl sulphate, 45% dimethylformamide) for 16~18 h. A550 nm and 650 nm (reference absorbance) were measured using a Model 680 Microplate Reader.

### 4.10. Western Blot

The immunoblotting procedures were performed as described previously [12,23]. In brief, treated cells were lysed in RIPA buffer (50 mM Tris (pH 7.4), 150 mM NaCl, 1% Nonidet P-40, 0.25% sodium deoxycholate, 5 mM EDTA (pH 8.0), and 1 mM EGTA (pH 8.0)) containing a protease inhibitor cocktail (Sigma-Aldrich) and quantitated using Bradford assays. An equivalent amount of cell lysate (50 μg) was analysed using SDS-PAGE with the appropriate antibodies. Images were quantified using Image J version 1.48 (NIH, Bethesda, MD, USA).

### 4.11. ROS Detection

RAW 264.7 cells (5 × 10^5^ cells in 6-well plates) were treated with pilloin, vehicle (0.1% DMSO) or andrographolide (positive control) for 1 h, followed by LPS (100 ng/mL) for 24 h. Cellular ROS production was measured by incubating treated cells with dichlorofluorescein diacetate (H_2_DCFDA, Invitrogen, Grand Island, NY, USA) at 10 μM in PBS for 30 min and then analysed using flow cytometry as described previously [43].

### 4.12. Phagocytosis Assay

Phagocytosis activity was measured using Vybrant Phagocytosis Assay Kit (Molecular Probes, Eugene, OR, USA). In brief, RAW 264.7 cells (2 × 10^5^ cells in 24-well plates) were treated with pilloin, fisetin (positive control) or vehicle (0.1% DMSO) for 24 h and incubated with 50 μg/mL of bioparticles (fluorescein-labelled *Escherichia coli*)) for 30 min. The supernatant was removed and then the cells were treated with Trypan Blue to quench the remaining extracellular bioparticles, washed with PBS, trypsinised and analysed under excitation/emission wavelengths of 480 nm/520 nm using Infinite^®^ 200 PRO (Tecan Group Ltd.). The relative phagocytic activity of the treated cells is determined according to the manufacturer’s protocol.

### 4.13. LPS-Induced Inflammatory Animal Model

Male C57BL/6 mice (10–12 weeks old) were purchased from the Animal Center of National Yang-Ming University (Taipei, Taiwan) and maintained in a specific pathogen-free area of this center. The endotoxin model was used to induce inflammation responses as described previously [44,45]. In brief, mice were divided into four groups for the following treatments: vehicle group (0.6% DMSO in 0.1% carboxymethyl cellulose (CMC)), pilloin group (10 mg/kg pilloin in vehicle + LPS 20 mg/kg), LPS group (vehicle + LPS 20 mg/kg) and pyrrolidine dithiocarbamate (PDTC) group (positive control; 50 mg/kg PDTC in vehicle + LPS 20 mg/kg). The vehicle or drug was intraperitoneally (i.p.) delivered into mice 1 h before LPS injection. After 12-h treatment, mice were sacrificed and the serum as well as tissues was collected for ELISA analysis. Serum glutamate oxaloacetate transaminase (GOT), glutamate pyruvate transaminase (GPT) and creatinine (CRE) were measured using the FUJI DRI-CHEM 4000i (Fujifilm Corp., Tokyo, Japan). The animal protocol was reviewed and approved by the Animal Care and Use Committee of National Yang Ming University (No. 1050902).

### 4.14. Statistical Analyses

Results are expressed as the mean ± SD from at least three independent experiments. The in vivo data are presented as the mean ± SEM. Comparisons between groups were performed using ANOVA followed by post hoc Dunnett’s test. A *p* value of <0.05 was considered statistically significant.

## Supplementary Materials

The following are available online. Figure S1: ESI-MS spectrum of pillion, Figure S2: ^1^H-NMR spectrum of pillion (CDCl_3_, 500 MHz), Figure S3: The effect of pilloin on serum biochemical markers in mice.

## References

[1] Karin M., Clevers H. (2016). Reparative inflammation takes charge of tissue regeneration. Nature.

[2] Wu X.-Q., Dai Y., Yang Y., Huang C., Meng X.-M., Wu B.-M., Li J. (2016). Emerging role of microRNAs in regulating macrophage activation and polarization in immune response and inflammation. Immunology.

[3] Ye H., Wang Y., Jenson A.B., Yan J. (2016). Identification of inflammatory factor TNF-α inhibitor from medicinal herbs. Exp. Mol. Pathol..

[4] Zhu L., Zhao Q., Yang T., Ding W., Zhao Y. (2015). Cellular metabolism and macrophage functional polarization. Int. Rev. Immunol..

[5] Lamkanfi M., Dixit V.M. (2012). Inflammasomes and their roles in health and disease. Annu. Rev. Cell Dev. Biol..

[6] Fink M.P., Warren H.S. (2014). Strategies to improve drug development for sepsis. Nat. Rev. Drug Discov..

[7] Baker R.G., Hayden M.S., Ghosh S. (2011). NF-κB, inflammation, and metabolic disease. Cell Metab..

[8] Schulert G.S., Grom A.A. (2015). Pathogenesis of macrophage activation syndrome and potential for cytokine-directed therapies. Annu. Rev. Med..

[9] Tak P.P., Firestein G.S. (2001). NF-κB: A key role in inflammatory diseases. J. Clin. Investig..

[10] Caamaño J., Hunter C.A. (2002). NF-κB family of transcription factors: Central regulators of innate and adaptive immune functions. Clin. Microbiol. Rev..

[11] Lawrence T. (2009). The nuclear factor NF-κB pathway in inflammation. Cold Spring Harb. Perspect Biol..

[12] Liu S.-H., Lin C.-H., Hung S.-K., Chou J.-H., Chi C.-W., Fu S.-L. (2010). Fisetin inhibits lipopolysaccharide-induced macrophage activation and dendritic cell maturation. J. Agric. Food Chem..

[13] Wu M.Y., Hung S.K., Fu S.L. (2011). Immunosuppressive effects of fisetin in ovalbumin-induced asthma through inhibition of NF-κB activity. J. Agric. Food Chem..

[14] Xia Y.F., Ye B.Q., Li Y.D., Wang J.G., He X.J., Lin X., Yao X., Ma D., Slungaard A., Hebbel R.P. (2004). Andrographolide attenuates inflammation by inhibition of NF-κB activation through covalent modification of reduced cysteine 62 of p50. J. Immunol..

[15] Lee K.C., Chang H.H., Chung Y.H., Lee T.Y. (2011). Andrographolide acts as an anti-inflammatory agent in LPS-stimulated RAW264.7 macrophages by inhibiting STAT3-mediated suppression of the NF-κB pathway. J. Ethnopharmacol..

[16] Hoensch H.P., Oertel R. (2015). The value of flavonoids for the human nutrition: Short review and perspectives. Clin. Nutri. Exp..

[17] Cheng J.T., Wu X.D., Li Y., Han Y.Q., Dong L.B., Zhao Q.S. (2013). Two new tirucallane triterpenoids from the leaves of *Aquilaria sinensis*. Arch. Pharm. Res..

[18] Ampofo S.A., Roussrs V., Wiemer D.F. (1987). New prenylated phenolics from *Piper auruum*. Phytochemistry.

[19] Shan J., Wang X., Ma Y., Yang R., Li X., Jin Y. (2010). Flavonoids from leaves of *Murraya panaculata* L. (I.). Zhongguo Yaoxue Zazhi.

[20] Liang S., Tian J.-M., Feng Y., Liu X.-H., Xiong Z., Zhang W.-D. (2011). Flavonoids from *Daphne aurantiaca* and their inhibitory activities against Nitric Oxide production. Chem. Pharm. Bull..

[21] Herz W., Sosa V.E. (1988). Sesquiterpene lactones and other constituents of *Arnica acaulis*. Phytochemistry.

[22] Nunez-Alarcon J. (1971). Pilloin, a new flavone from *Ovidia Pillo-Pillo*. J. Org. Chem..

[23] Fu S.L., Hsu Y.H., Lee P.Y., Hou W.C., Hung L.C., Lin C.H., Chen C.M., Huang Y.J. (2006). Dioscorin isolated from *Dioscorea alata* activates TLR4-signaling pathways and induces cytokine expression in macrophages. Biochem. Biophys. Res. Commun..

[24] Linehan E., Dombrowski Y., Snoddy R., Fallon P.G., Kissenpfennig A., Fitzgerald C.D. (2014). Aging impairs peritoneal but not bone marrow-derived macrophage phagocytosis. Aging Cell.

[25] Arthur J.S.C., Ley S.C. (2013). Mitogen-activated protein kinases in innate immunity. Nat. Rev. Immunol..

[26] Chaudhry H., Zhou J., Zhong Y., Ali M.M., Mcguire F., Nagarkatti P.S., Nagarkatti M. (2013). Role of cytokines as a double-edged sword in sepsis. In Vivo.

[27] Cohen J. (2002). The immunopathogenesis of sepsis. Nature.

[28] Matsuda H., Morikawa T., Ando S., Toguchida I., Yoshikawa M. (2003). Structural requirements of flavonoids for nitric oxide production inhibitory activity and mechanism of action. Bioorg. Med. Chem..

[29] Grael C.F.F., Kanashiro A., Kabeya L.M., Jordão C.O., Takeara R., Gobbo-Neto L., Polizello A.C.M., Lucisano-Valim Y.M., Lopes N.P.L., Lopes J.L.C. (2010). In vitro study of antioxidant and scavenger properties of phenolic cpmpounds from Lychnophora species. Quim Nova.

[30] Akira S., Uematsu S., Takeuchi O. (2006). Pathogen recognition and innate immunity. Cell.

[31] Matsuda N., Hattori Y. (2006). Systemic inflammatory response syndrome (SIRS): Molecular pathophysiology and gene therapy. J. Pharmacol. Sci..

[32] Lu Y.C., Yeh W.C., Ohashi P.S. (2008). LPS/TLR4 signal transduction pathway. Cytokine.

[33] Barnes P.J., Adcock I.M. (1997). NF-κB: A pivotal role in asthma and a new target for therapy. Trends Pharmacol. Sci..

[34] Guha M., Mackman N. (2001). LPS induction of gene expression in human monocytes. Cell. Signal..

[35] Ahmed K.M., Dong S., Fan M., Li J.J. (2006). Nuclear factor-kappaB p65 inhibits mitogen-activated protein kinase signaling pathway in radioresistant breast cancer cells. Mol. Cancer Res..

[36] Chen C., Chen Y.H., Lin W.W. (1999). Involvement of p38 mitogen-activated protein kinase in lipopolysaccharide-induced iNOS and COX-2 expression in J774 macrophages. Immunology.

[37] Boomer J.S., Green J.M., Hotchkiss R.S. (2014). The changing immune system in sepsis: Is individualized immuno-modulatory therapy the answer?. Virulence.

[38] Lago J.H.G., Toledo-Arruda A.C., Mernak M., Barrosa K.H., Martins M.A., Tibério I.F.L.C., Prado C.M. (2014). Structure-activity association of flavonoids in lung diseases. Molecules.

[39] Walle T. (2009). Methylation of dietary flavones increases their metabolic stability and chemopreventive effects. Int. J. Mol. Sci..

[40] Michelis F., Tiligada E., Skaltsa H., Lazari D., Skaltsounis A.-L., Delitheos A. (2002). Effects of the flavonoid pilloin Isolated from *Marrubium cylleneum* on mitogen-induced lymphocyte transformation. Pharm. Biol..

[41] Wang S.L., Hwang T.L., Chung M.I., Sung P.J., Shu C.W., Cheng M.J., Chen J.J. (2015). New Flavones, a 2-(2-Phenylethyl)-4*H*-chromen-4-one derivative, and anti-inflammatory constituents from the stem barks of *Aquilaria sinensis*. Molecules.

[42] Sun J., Zhang X., Broderick M., Fein H. (2003). Measurement of Nitric Oxide production in biological systems by using Griess reaction assay. Sensors.

[43] Liu S.H., Lin C.H., Liang F.P., Chen P.F., Kuo C.D., Alam M.M., Mait B., Hung S.K., Chi C.W., Sun C.M. (2014). Andrographolide downregulates the v-Src and Bcr-Abl oncoproteins and induces Hsp90 cleavage in the ROS-dependent suppression of cancer malignancy. Biochem. Pharmacol..

[44] Fink M.P. (2014). Animal models of sepsis. Virulence.

[45] Yuk J.M., Shin D.M., Lee H.M., Kim J.J., Kim S.W., Jin H.S., Yang C.S., Park K.A., Chanda D., Kim D.K. (2011). The orphan nuclear receptor SHP acts as a negative regulator in inflammatory signaling triggered by Toll-like receptors. Nat. Immunol..

