# Supplementary material for: Pilloin, A Flavonoid Isolated from Aquilaria sinensis, Exhibits Anti-Inflammatory Activity In Vitro and In Vivo
Source: Molecules. 2018 Dec 2;23(12):3177. doi: 10.3390/molecules23123177 (PMC6320910; doi:10.3390/molecules23123177)
Supplement: Supplementary file 1 [file molecules-23-03177-s001.pdf]

## Supplementary Information

# Pilloin, A Flavonoid Isolated from *Aquilaria sinensis*, Exhibits Anti-Inflammatory Activity In Vitro and In Vivo

**Yun-Chen Tsai [1], Sin-Ling Wang [2], Mei-Yao Wu [3], Chia-Huei Liao [1], Chao-Hsiung Lin [4], Jih-Jung Chen [5,6,*] and Shu-Ling Fu [1,*]**

[1] Institute of Traditional Medicine, National Yang-Ming University, Taipei 11221, Taiwan; tyc202006@gmail.com (Y.-C.T.); sweet791020@gmail.com (C.-H.L.)
[2] School of Pharmacy, College of Pharmacy, Kaohsiung Medical University, Kaohsiung 80708, Taiwan; s8332805@yahoo.com.tw
[3] Research Center for Traditional Chinese Medicine, Department of Medical Research, China Medical University Hospital, Taichung 40402, Taiwan; meiyao19790919@yahoo.com.tw
[4] Department of Life Sciences and Institute of Genome Sciences, National Yang-Ming University, Taipei 11221, Taiwan; chlin2@ym.edu.tw
[5] Faculty of Pharmacy, National Yang-Ming University, Taipei 11221, Taiwan
[6] Department of Medical Research, China Medical University Hospital, China Medical University, Taichung 40447, Taiwan
* Correspondence: chenjj@ym.edu.tw (J.-J.C.); slfu@ym.edu.tw (S.-L.F.); Tel.: +886-2-28267000 (ext. 7195) (J.-J.C.); +886-2-28267177 (S.-L.F.); Fax: +886-2-28232940 (J.-J.C.); +886-2-28225044 (S.-L.F.)

**Figure 1.** ESI-MS spectrum of pillion.

**Figure 2.** $^1$H-NMR spectrum of pillion (CDCl3, 500 MHz).

**Figure 3.** The effect of pilloin on serum biochemical markers in mice.

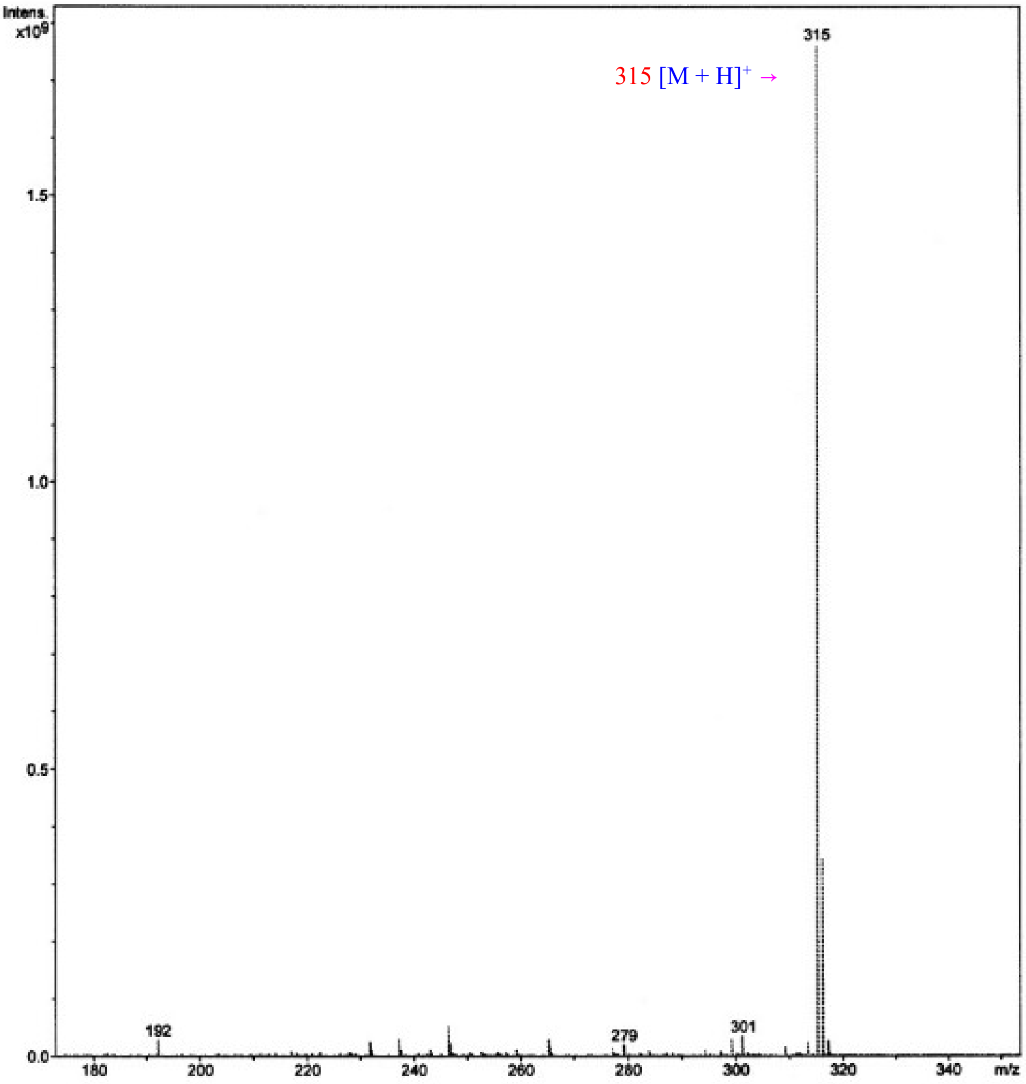


**Figure 1.** ESI-MS spectrum of pillion.

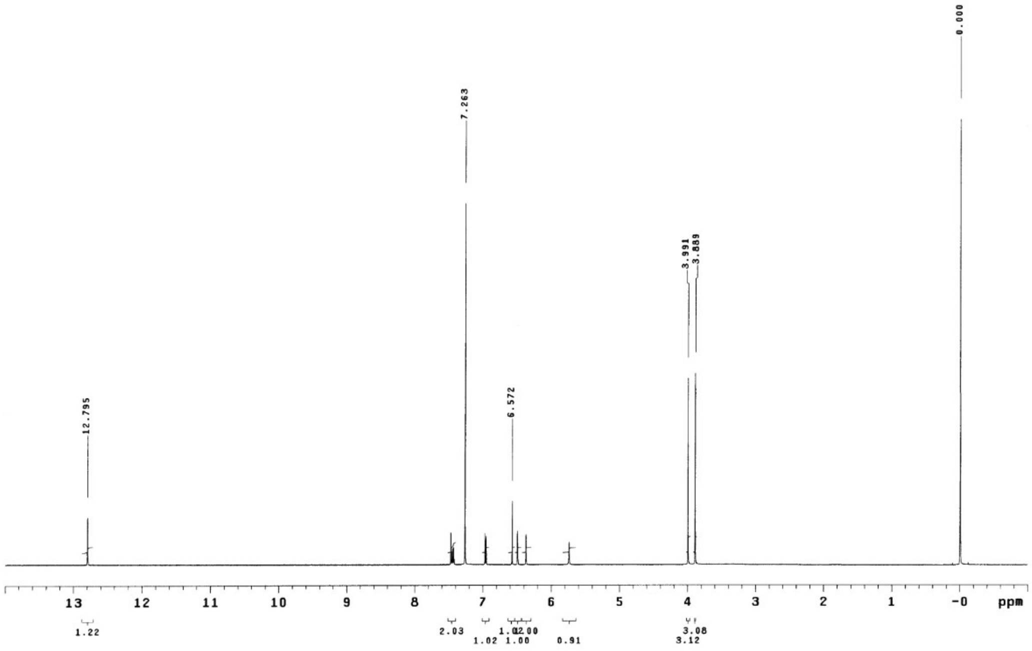


**Figure 2.** ¹H-NMR spectrum of pillion (CDCl3, 500 MHz).

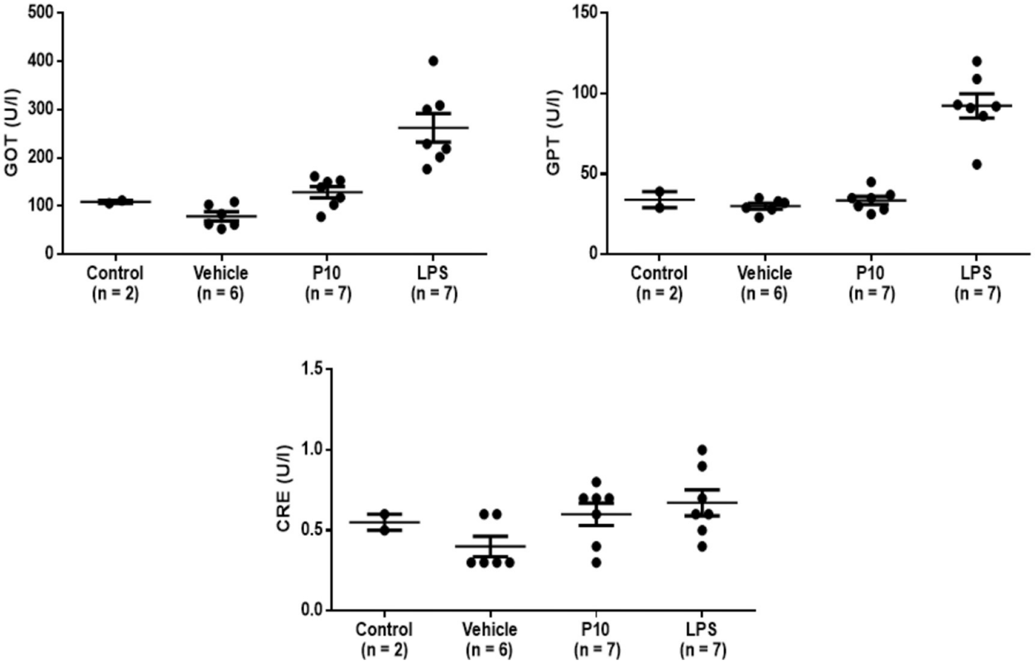


**Figure 3.** The effect of pilloin on serum biochemical markers in mice. Male C57BL/6 mice (10~12 weeks old) were intraperitoneally injected with vehicle or drug. The serum biochemical data were collected from mice exposed to various treatments for 13 h. GOT, GPT and creatinine (CRE) were measured using the FUJI DRI-CHEM 4000i (Fujifilm Corp., Tokyo, Japan). The values are shown as means ± SEM. control: untreated mice; vehicle: 0.6% DMSO in 0.1% carboxymethyl cellulose; LPS: 20 mg/kg lipopolysaccharide; P10: 10 mg/kg pilloin.
